# 2683. Follow-up Testing for *Chlamydia trachomatis* and *Neisseria gonorrhea* Infections in Female Basic Military Trainees

**DOI:** 10.1093/ofid/ofad500.2294

**Published:** 2023-11-27

**Authors:** Lisa Townsend, Shauna Stahlman, Gitaik Oh, James Escobar, Angela Osuna, Theresa Casey, Erin Winkler, John Kieffer, Heather Yun, Jason F Okulicz, Joseph Marcus

**Affiliations:** San Antonio Uniformed Services Health Education Consortium, San Antonio, Texas; Armed Forces Health Surveillance Division, Silver Spring, Maryland; DHA Health Surveillance, Silver Spring, Maryland; USAF Epidemiology Consult Service Division, Wright-Patterson Air Force Base, Ohio; BAMC, San Antonio, Texas; BAMC, San Antonio, Texas; BAMC, San Antonio, Texas; BAMC, San Antonio, Texas; Brooke Army Medical Center, TX; San Antonio Military Medical Center, San Antonio, Texas; Brooke Army Medical Center, TX

## Abstract

**Background:**

The Centers for Disease Control and Prevention recommends follow-up testing for *Chlamydia trachomatis* (CT) and *Neisseria gonorrhea* (GC) infections 3 months after initial positive test or within one year if repeat testing is not possible at 3 months. Follow-up testing compliance in the United States is incompletely understood and has been reported below 35%, even in universally insured populations. The rates of follow-up testing in military populations are unknown.

**Methods:**

During the study period from January 1, 2006-December 31, 2021, all female Air Force Basic Military Trainees provided a urine sample which underwent nucleic acid amplification testing (NAAT) for CT and GC. Those who tested positive were evaluated to determine demographic information as well as follow-up testing rates. Patients who were evaluated with a repeat CT/GC NAAT test within 12 months after a positive CT/GC test result were considered to have received appropriate follow-up. Nominal variables were compared by the Chi-squared test. A p-value of < 0.05 was considered significant.

**Results:**

A total of 97,168 women were tested over the 16-year study period. Of those tested, 4,687 (4.8%) tested positive for either CT (n=4,584, 94.5%), GC (n=134, 2.7%), or both (n=131, n=2.7%). Of those who tested positive, 3,268 (69.7%) had repeat testing within 12 months of their initial positive test. There was no significant difference in follow-up by age, race, educational level, marital status, or organism. Compared to 2006-2009, 2018-2021 had significantly more follow-up testing (66.0% vs. 77.7%, p=< 0.0001) (**Figure**).

Follow-up Testing Rates for Chlamydia trachomatis and Neisseria gonorrhea Infections in Female Basic Military Trainees 2006-2021

**Figure:**
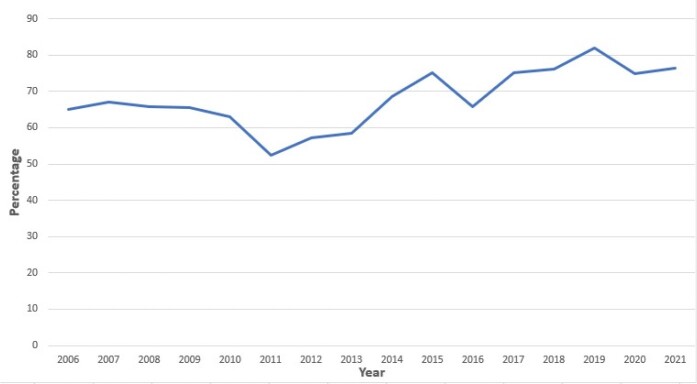

**Conclusion:**

Follow-up testing among female Air Force Basic Military Trainees is significantly higher than in other universally insured populations and has been improving with time. However, this study shows remaining barriers to adherence to recommended guidelines. Future efforts should clarify remaining barriers to follow-up testing in both military and civilian populations.

**Disclosures:**

**Jason F. Okulicz, MD**, Gilead Sciences: Advisor/Consultant

